# Cryo-EM reconstruction of the chlororibosome to 3.2 Å resolution within 24 h

**DOI:** 10.1107/S205225251701226X

**Published:** 2017-09-22

**Authors:** Björn O. Forsberg, Shintaro Aibara, Dari Kimanius, Bijoya Paul, Erik Lindahl, Alexey Amunts

**Affiliations:** a Science for Life Laboratory, Department of Biochemistry and Biophysics, Stockholm University, 171 65 Solna, Sweden

**Keywords:** cryo-EM, image processing, chlororibosome

## Abstract

The introduction of direct detectors and the automation of data collection in cryo-EM have led to a surge in data, creating new opportunities for advancing computational processing. In particular, on-the-fly workflows that connect data collection with three-dimensional reconstruction would be valuable for more efficient use of cryo-EM and its application as a sample-screening tool. Here, accelerated on-the-fly analysis is reported with optimized organization of the data-processing tools, image acquisition and particle alignment that make it possible to reconstruct the three-dimensional density of the 70S chlororibosome to 3.2 Å resolution within 24 h of tissue harvesting. It is also shown that it is possible to achieve even faster processing at comparable quality by imposing some limits to data use, as illustrated by a 3.7 Å resolution map that was obtained in only 80 min on a desktop computer. These on-the-fly methods can be employed as an assessment of data quality from small samples and extended to high-throughput approaches.

## Abstract 2

The organization of cryo-EM image-processing tools can be tailored for acceleration of the data analysis. Here, this is exemplified by the cryo-EM structure determination of the chlororibosome from spinach leaves to 3.2 Å resolution within 24 h.

## 1. Introduction   

The range of biological macromolecular structures that have been determined to high resolution by cryo-EM has expanded tremendously in the last three years (Kühlbrandt, 2014 ▸; Fernandez-Leiro & Scheres, 2016 ▸; Subramaniam *et al.*, 2016 ▸). This expansion has been paralleled by a rapid growth in the cryo-EM research community, as numerous research centres acquire cutting-edge infrastructures with high-end microscopes and centrally funded cryo-EM facilities are established (Stuart *et al.*, 2016 ▸). Substantial amounts of data are now collected using increasingly automated modes of operation (Tan *et al.*, 2016 ▸), whereas the availability of advanced statistical methods employing maximum-likelihood approaches allows even non-experts to analyse heterogeneous populations of particles (Amunts *et al.*, 2014 ▸; Scheres *et al.*, 2007 ▸; Grigorieff, 2016 ▸; Kimanius *et al.*, 2016 ▸; de la Rosa-Trevín *et al.*, 2016 ▸). Hence, new challenges have emerged of how to maximize the efficiency of data acquisition and subsequently extract the structural information.

Recently, models of the chloroplast 70S ribosome prepared from spinach leaves have been reported to 3.4–5.4 Å resolution (Bieri *et al.*, 2017 ▸; Ahmed *et al.*, 2016 ▸; Graf *et al.*, 2017 ▸). Here, we examine how accelerated processing can be used for well behaved complexes such as the chlororibosome, and suggest settings that might be employed as a high-throughput approach. Particularly, we describe accelerated data processing workflows, using which a 3.2 Å resolution map of the chlororibosome can be achieved within 24 h from tissue harvest and a 3.7 Å resolution map can be calculated within 80 min of processing.

## 2. Methods   

### 2.1. Purification of chlororibosomes   

Seven spinach leaves were blended for 1 min in a buffer consisting of 0.7 *M* sorbitol, 10 m*M* Tris–HCl pH 7.6, 50 m*M* KCl and 5 m*M* magnesium acetate. The suspension was filtered through cheesecloth and spun down twice at 1200*g* for 15 min. The cells were disrupted by the addition of 2% Triton X-100 and the membranes were separated by centrifugation at 16 000*g* for 20 min. Crude chlororibosomes were precipitated by the addition of 8% PEG 10 000 and collected at 12 000*g* for 10 min. The pellet was resuspended in 1 ml of the same buffer but with 0.4 *M* sorbitol and loaded onto a 0.5 ml cushion of the same buffer but with 1 *M* sucrose. The sample was centrifuged at 230 000*g* for 15 min, and the pellet was resuspended, loaded onto a 15–30% sucrose gradient in buffer consisting of 25 m*M* HEPES–KOH pH 7.5, 50 m*M* KCl, 10 m*M* magnesium acetate and centrifuged at 213 000*g* for 90 min using a TLS-55 rotor. Fractions corresponding to intact chlororibosomes, putative subunits and additional higher molecular-weight contaminants were pooled and sucrose was removed by buffer exchange.

### 2.2. Cryo-EM and image processing   

3 µl of purified chlororibosomes were applied onto freshly glow-discharged Quantifoil R2/2 grids coated with a thin layer of continuous carbon (∼3 nm thick) at 4°C and 100% humidity. After 30 s incubation, the grids were blotted for 3 s and vitrified by plunging into liquid ethane using a Vitrobot Mark IV (FEI) system. Images were acquired using automated acquisition software (*EPU* from FEI) on a Titan Krios microscope (FEI) operating at 300 kV using a Falcon II direct electron detector at a magnification of 1.06 Å per pixel. Dose-fractionated movies were acquired over 1.5 s (25 frames per movie) at a dose rate of ∼19 e^−^ Å^−2^ at a nominal magnification of 75 000×, yielding a calibrated pixel size of 1.06 Å, on a Falcon II direct electron detector (FEI). The final data set at the point of processing contained a defocus range of between 0.25 and 3.26 µm. Data were processed using a Linux workstation equipped with an eight-core Intel Core i7-5960x CPU and four GPUs. All of the 24 h time trials and reconstructions from the original data were performed with consumer GPUs (NVIDIA GTX 1080). Some of the rescaled images were processed when the same machine had been fitted with GP100 GPUs; for identical data the performance difference between the cards was below 2%.

The computational load was decreased by limiting the number of orientations that were selected to be more finely sampled in the second pass of alignment in *RELION*-2.0. During the two-dimensional classification, the limit was set to 10 using the option --maxsig 10. In order to use a subset of 30% of the full input data for the first five iterations, the options --subset_iter 5 --subset_frac 0.3 were used. The two-dimensional classification resulted in 100 475 particles that were extracted with 420 × 420 pixel boxes. The initial model for three-dimensional refinement was generated using a low-pass map filtered to 60 Å.

Reported resolutions are based on the gold-standard FSC = 0.143 criterion unless otherwise stated, and FSC curves were corrected for the effects of a soft mask on the FSC curve using high-resolution noise substitution (Chen *et al.*, 2013 ▸).

## 3. Results   

**Figure 1:**
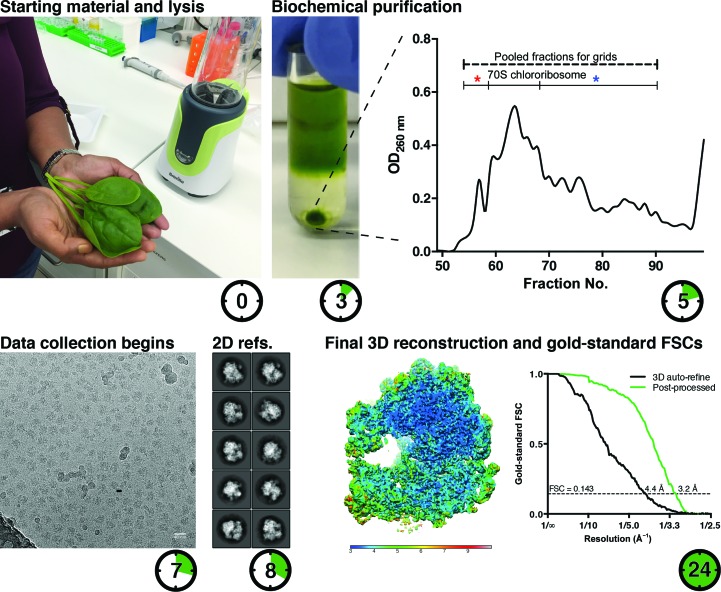
Cryo-EM structure determination of the chlororibosome to 3.2 Å resolution within 24 h. The experiment started with seven spinach leaves and chlororibosomes were purified within 5 h, appearing as a major peak in the sucrose gradient. Contaminating fractions labelled with asterisks were included for grid preparation. Data collection started after 7 h, and micrographs exhibited ice contamination owing to high humidity conditions during grid handling. The data were processed on-the-fly, resulting in a 3.2 Å resolution reconstruction after 24 h.

To obtain a reconstruction of the chlororibosome, seven spinach leaves were subjected to a biochemical purification protocol including blending, PEG precipitation and sucrose cushion and gradient, which were completed within 5 h (Fig. 1 ▸). Grids were deliberately prepared with the inclusion of fractions representing dissociated chlororibosomes and higher molecular-weight contaminants (Fig. 1 ▸), in order to represent a non-ideal sample, and ice contamination was also present (Supplementary Fig. S1). The cryo-EM sample preparation, grid screening and setup of automated data-collection parameters were performed within 1 h. However, it is likely to take longer for less optimized samples. A total of 865 micrographs were collected using an FEI Titan Krios microscope equipped with a Falcon II detector (1.06 Å per pixel) during a 10 h session. Micrographs were processed on-the-fly using a desktop Linux workstation equipped with four NVIDIA GTX 1080 graphics cards, finishing just over 24 h after the biochemical protocol was initiated. The reconstructed map then showed a resolution of 3.2 Å.

**Figure 2:**
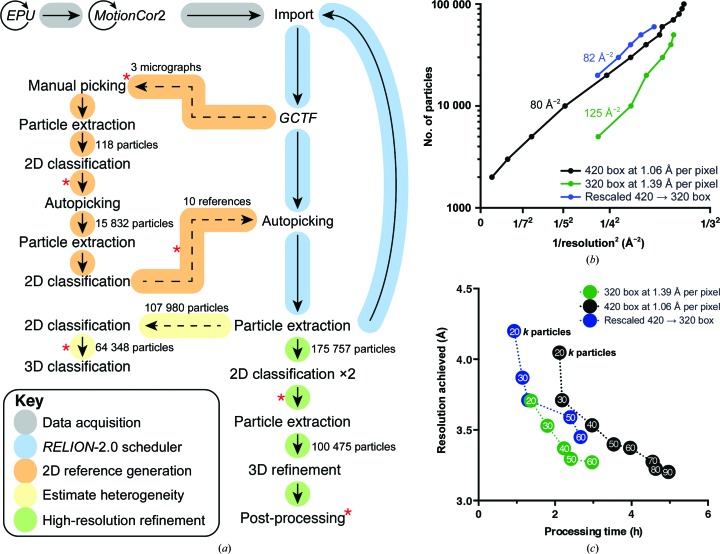
Data-processing workflow and correlation between data-collection parameters, processing time and resolution. (*a*) Data-processing workflow. (*b*, *c*) Effect of particle number and processing time on attained resolution at magnifications corresponding to 1.06 and 1.39 Å per pixel. Steps in which manual intervention is presently required are marked by red asterisks. Note, however, that the scheduling cycle does not rely on any such intervention.

The data-processing workflow is depicted in Fig. 2 ▸(*a*), and FSC curves of the obtained maps are presented in Supplementary Fig. S4. Alignment and dose-weighting of raw movies were performed using *UcsfDfCorr* (*MotionCor*2; Zheng *et al.*, 2017 ▸) with a 5 × 5 patch size, contrast transfer function parameters were estimated using *GCTF* (Zhang, 2016 ▸), and image processing was conducted within the *RELION*-2.0 GUI interface, heavily utilizing the scheduler function (Fernandez-Leiro & Scheres, 2016 ▸). 118 particles were manually identified from the first three micrographs and extracted with a box size fourfold downscaled to 100 pixels (Fig. 2 ▸ *a*, Supplementary Fig. S1). These were used for reference-free two-dimensional classification, which yielded initial class averages for automated particle picking with *RELION*-2.0. Particles were then automatically picked from the initial subset of 102 micrographs and again extracted with a fourfold reduced size, and subjected to two-dimensional classification with an upper limit of ten orientations considered in the oversampled alignment Bayesian approach in *RELION*. For the first five iterations, only a subset of the full input of the data was used (controlled with --subset_iter 5 --subset_frac 0.3), as initial convergence depends on neither high resolution nor large particle numbers. This allowed a large number of redundant calculations at low resolution to be avoided. From the two-dimensional classification results, ten representative class averages were selected that displayed distinct ribosome views (Supplementary Fig. S1). These classes were used for all subsequent rounds of automated particle picking.

Two-dimensional classification estimated that ∼10% of the picked particles represent ice contamination (Supplementary Fig. S2). A second round of two-dimensional classification was performed after excluding these, which resulted in improved class averages and revealed detailed structural features of the chlororibosome (Supplementary Fig. S2). Well resolved classes were then selected and subjected to three-dimensional refinement. The reconstruction was refined to a nominal resolution of 3.2 Å (Fig. 1 ▸, Supplementary Figs. S4 and S5). During data acquisition, three-dimensional classification was performed using fractional data and the restricted alignment described above. This was still found to be sufficiently accurate to reveal the presence of the chlororibosomal large subunit and contamination from cytoplasmic ribosomes, demonstrating that highly accelerated data processing is sufficient for the identification of even minor populations. Post-refinement three-dimensional classification showed the presence of the chlororibosomal large subunit (8%) and of the cytoplasmic ribosome (4%) (Supplementary Fig. S3).

To investigate the correlation between the results and the amount of acquired data, we refined just 500 particles from three micrographs, which still assigned ∼85% of particle orientations to within 4° of those obtained by using 50 000 particles (Supplementary Fig. S6). This corroborates the observation that *RELION* iterative reconstruction needs little data to distinguish fundamental objects. In the present case, the transition point below which it is no longer possible to relate particles to one another appears to be well below just a few hundred particles. This implies that statistical inferences about data quality are justified, and that data sets could potentially be characterized and assessed in very early stages using preliminary and small data sets. However, it is worth mentioning that while the gold-standard (GS) refinement using 500 particles reaches ∼25 Å (0.43 GS FSC criterion), it agrees to within 12 Å (0.5 FSC criterion) with a reconstruction utilizing identical particles with orientational assignments determined from the refinement of a larger data set with 50 000 particles. Evidently, signal statistics or possibly some other factor dominates the reconstruction error for smaller data sets, as opposed to resolution-dependent orientation inaccuracies.

To further explore the optimization of the data-processing properties, we systematically examined the reduction of calculation time as a function of the number of particles and evaluated its effects on the attained resolution (Figs. 2 ▸ *b* and 2 ▸ *c*). The results show that a 3.7 Å resolution reconstruction can be calculated by reducing the data to 30 000 particles (Fig. 2 ▸ *c*), which required 2 h of processing. Further restricting the data set to 20 000 particles limited the final resolution to 4.1 Å, but did not reduce the processing time, since the processing costs at this point are dominated by steps proportional to the image sizes rather than number of images used.

To examine the effect of increased pixel size on processing speed, we rescaled images from 1.06 to 1.39 Å per pixel. The consequent refinement of the same 20 000 particles led to a 4.2 Å reconstruction within 56 min, revealing the majority of the secondary-structure elements. Thus, rescaling images to obtain preliminary reconstruction during a microscope session can be a useful approach in some cases. Following the same rationale, we collected a new data set at 1.39 Å per pixel (Figs. 2 ▸ *b* and 2 ▸ *c*). A data set of 20 000 particles processed as before now yielded a 3.7 Å reconstruction of the chlororibosome with 80 min. The higher resolution compared with *in silico* rescaling is attributed to a better signal-to-noise ratio of the spatial frequencies most relevant to the refined resolution. The slightly longer processing time is caused by the increased number of iterations conducted during refinement to higher resolution. In addition, owing to the increased number of particles fitting in a micrograph with decreasing magnification, the data-collection time is also reduced. Thus, to reach a maximum throughput it is important for a user to carefully choose the weighting factors relating to transfer functions of the microscope and data-set quality.

Overall, the results show that the GPU-accelerated image processing and on-the-fly workflows allow rapid diagnosis of the specimen as data are acquired. When selecting parameters for the magnification and the number of images to collect and process, it is possible to reduce both microscope time and user effort by considering what practical resolution to target and the requirements posed by a biological question (Henderson, 2004 ▸). These considerations, enabling more rapid feedback, will be particularly useful for data evaluation and the determination of bound cofactors. The most recent software developments employing stochastic gradients to speed up *ab initio* model building (*cryoSPARC*; Punjani *et al.*, 2017 ▸), performing image-processing tasks while new data are being acquired at the microscope (*Focus*; Biyani *et al.*, 2017 ▸), incorporating different image processing into a single framework (*Scipion*; de la Rosa-Trevín *et al.*, 2016 ▸) and using a pipeline approach (Fernandez-Leiro & Scheres, 2017 ▸) will further improve the integration of experimental and computational parts of cryo-EM into a single setup in a user-friendly manner.

## 4. Accession codes   

The cryo-EM maps have been deposited in the Electron Microscopy Data Bank with accession codes EMD-3806, EMD-3807 and EMD-3808.

## Supplementary Material

Supplementary Figures.. DOI: 10.1107/S205225251701226X/kf5004sup1.pdf
