# Supplementary material for: Cryo-EM reconstruction of the chlororibosome to 3.2 Å resolution within 24 h
Source: IUCrJ. 2017 Sep 22;4(Pt 6):723–7. doi: 10.1107/S205225251701226X (PMC5668856; doi:10.1107/S205225251701226X)
Supplement: Supplementary file 1 [file m-04-00723-sup1.pdf]

IUCrJ

Volume 4 (2017)

Supporting information for article:

Cryo-EM reconstruction of the chlororibosome to 3.2 Å within 24 hours

Björn O. Forsberg, Shintaro Aibara, Dari Kimanius, Bijoya Paul, Erik Lindahl and Alexey Amunts

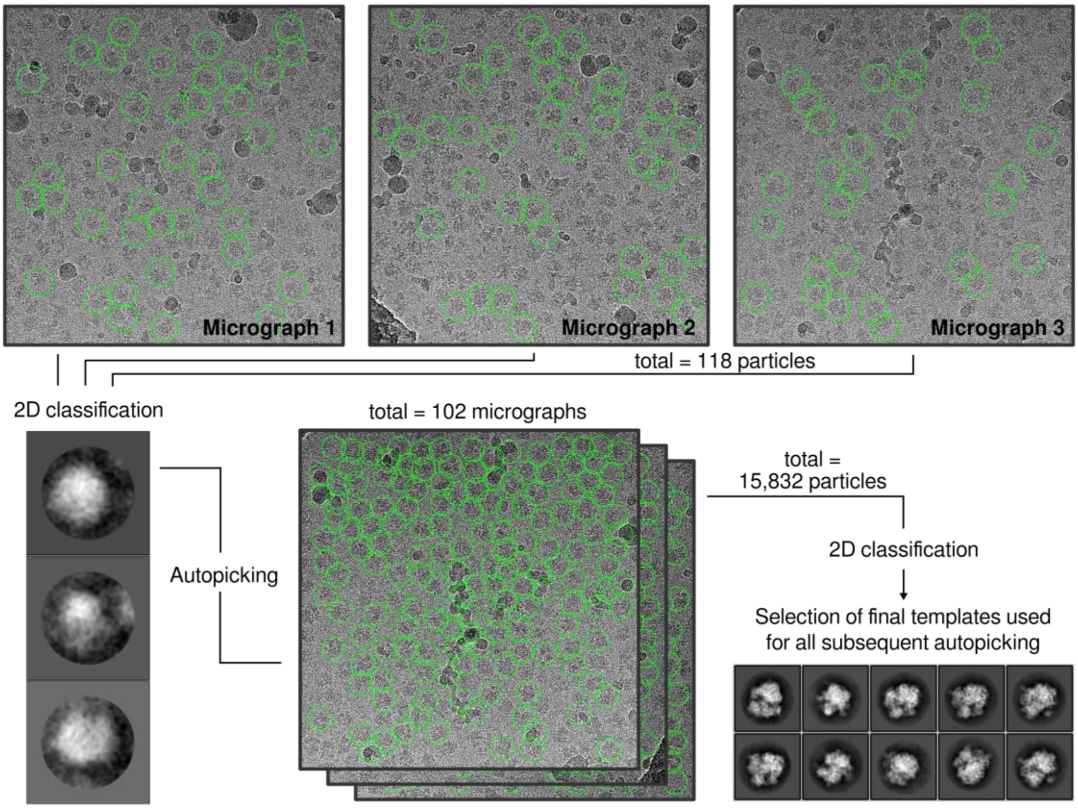

Figure S1 Selection of templates used for autopicking. 118 particles from the first three micrographs were used for reference-free 2D classification, yielding initial class-averages for autopicking from a 102-micrograph subset. Extracted particles, with a fourfold reduced size, were subjected to 2D classification according to the text, and ten representative class averages were selected for subsequent processing.

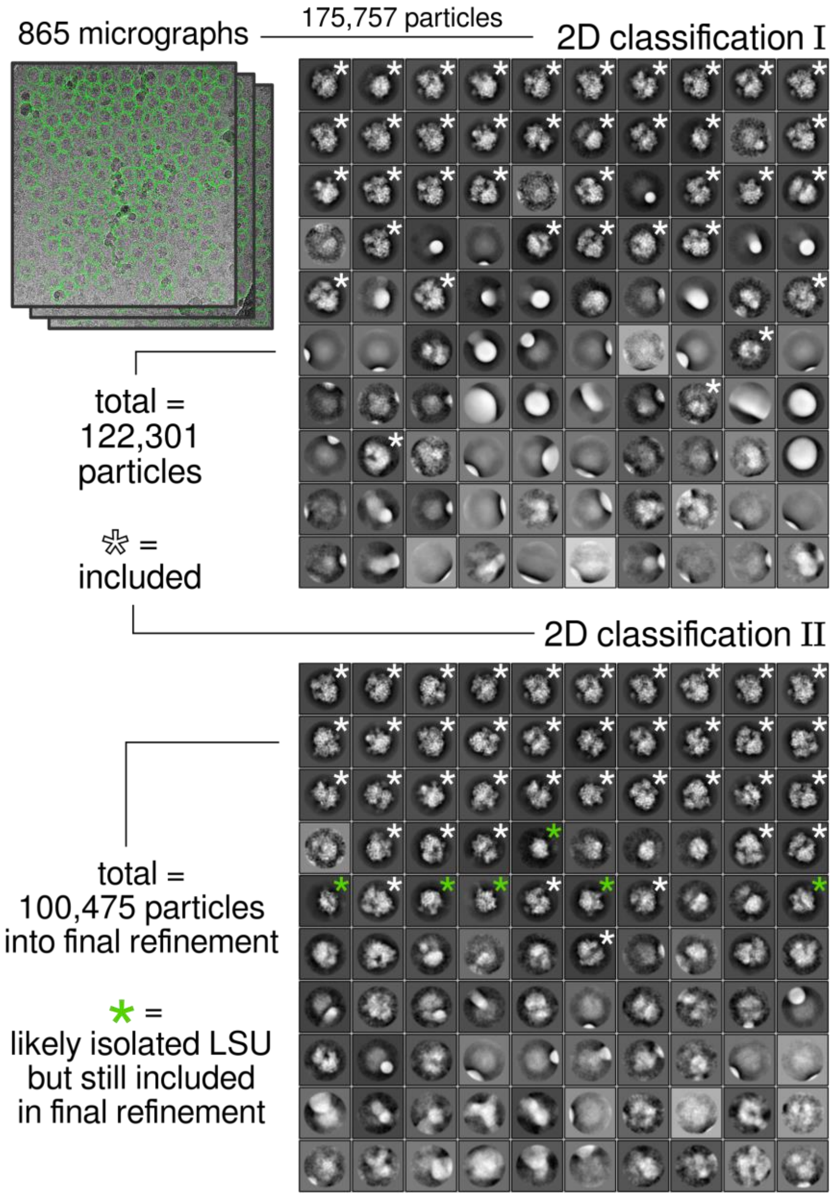

Figure S2 2D classification. The first round of 2D class averages showed substantial ice contaminations. A second round of 2D classification was performed after excluding these, which revealed detailed structural features of the chlororibosome.

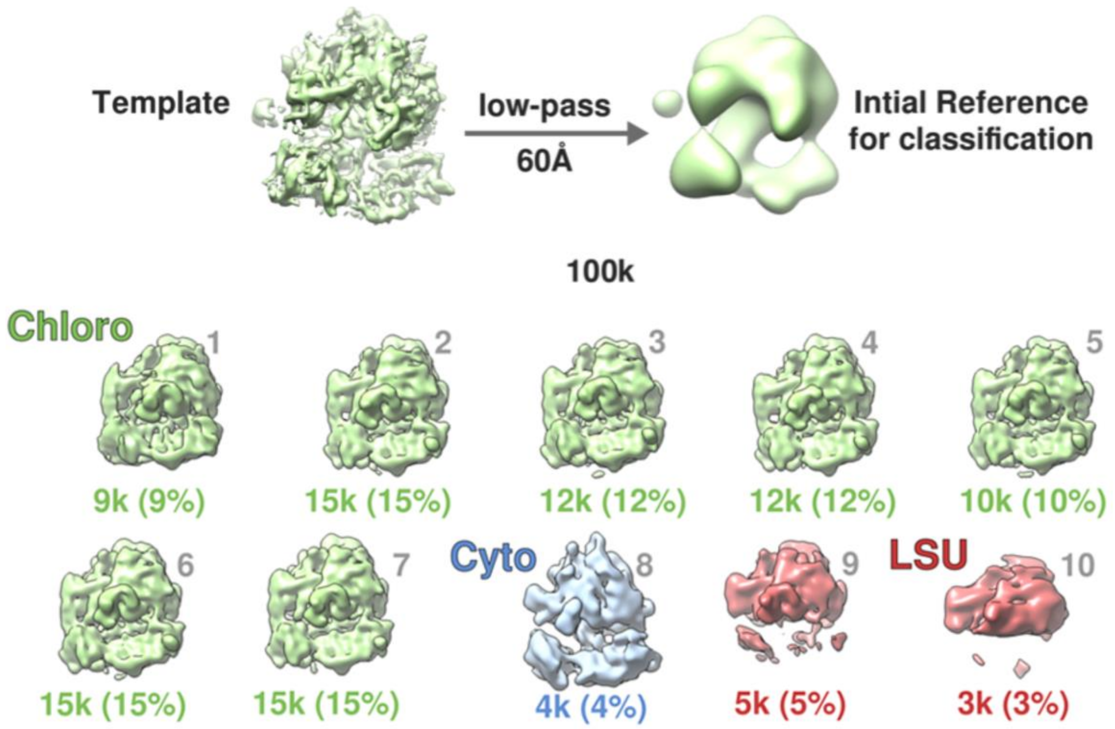

Figure S3 3D classification identifies minor populations. The full dataset was subjected to 3D classification that identified presence of the chlororibosomal large subunit (LSU) and cytoplasmic ribosomes, showing that accelerated processing is sensitive enough to identify subpopulations.

A

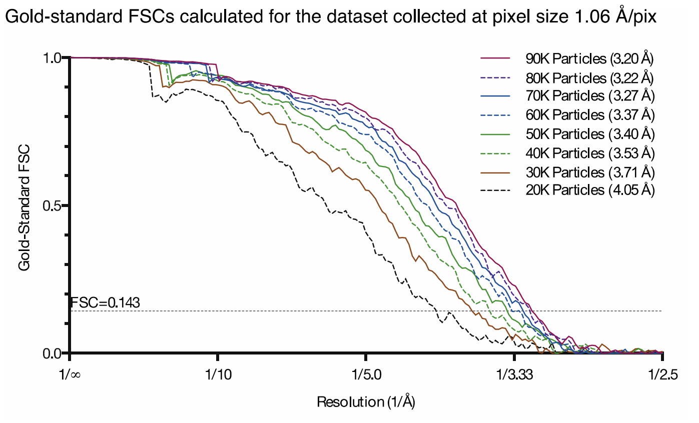

B

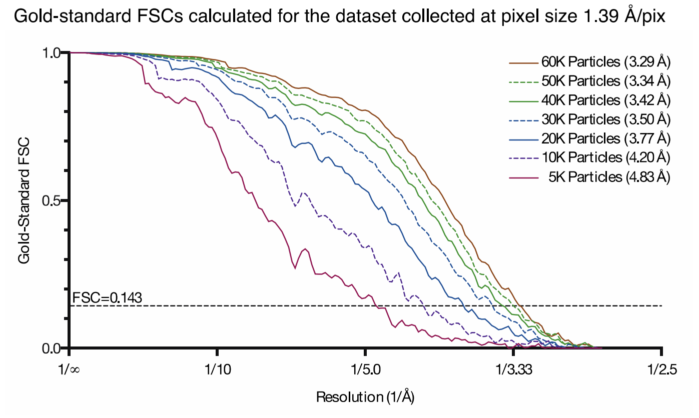

Figure S4 Fourier shell correlation curves. Resolution of the calculated maps reported according to the gold standard FSC=0.143 criterion as implemented in RELION.

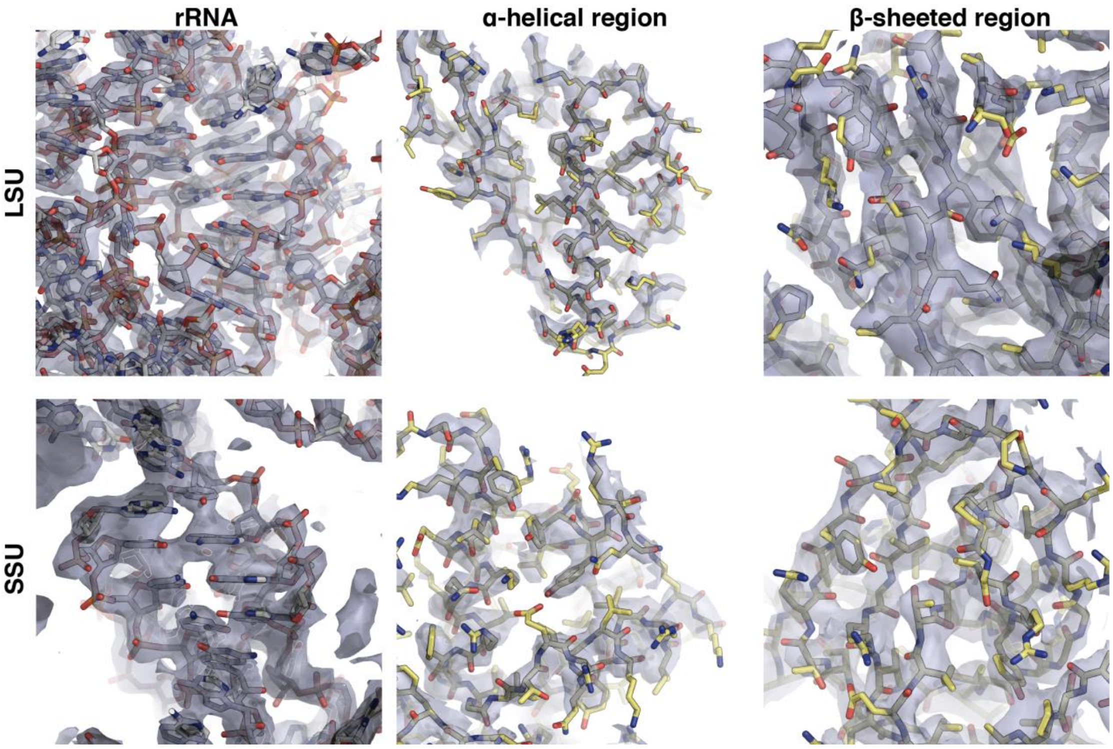

Figure S5 Sample views of different regions of the on-the-fly calculated map.

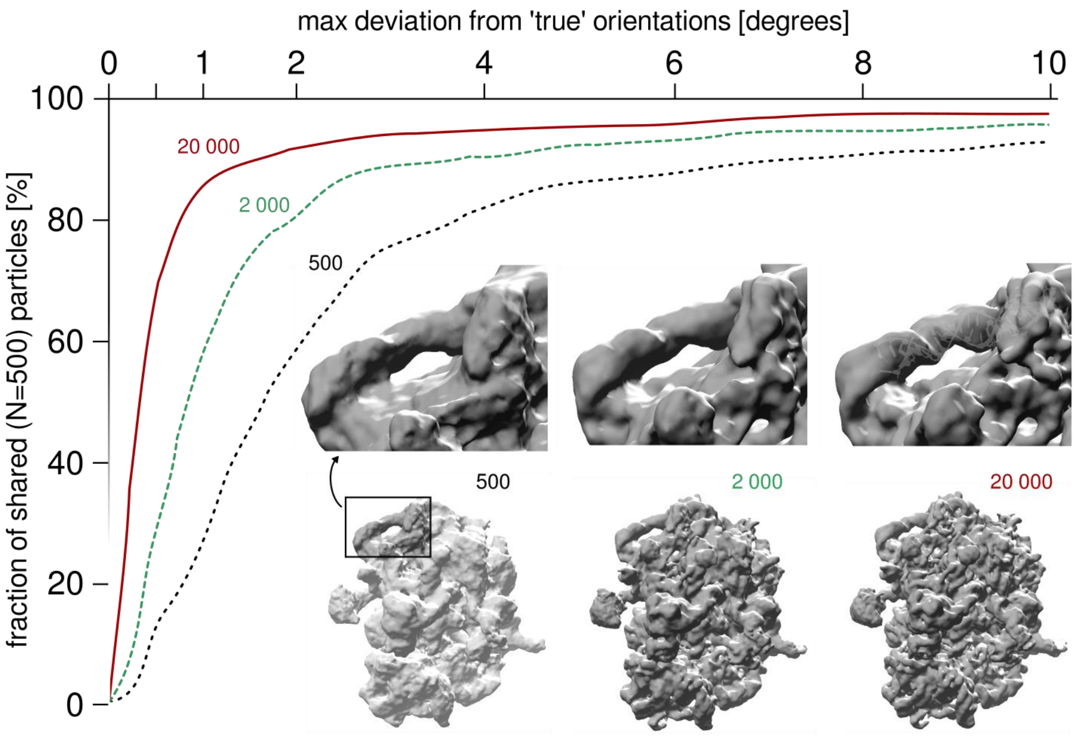

Figure S6 Alignment accuracies of different size datasets, as determined by distance on the unit sphere. The angle is measured using the first to Euler angles of the image orientation, which determine the geodesic distance between orientations on the unit sphere. In all cases the distance is determined against orientations of the identical 500 particles, as refined when part of a much larger dataset of 60k particles.
